# Supplementary material for: Landscape features support natural pest control and farm income when pesticide application is reduced
Source: Nat Commun. 2024 Jun 25;15:5384. doi: 10.1038/s41467-024-48311-3 (PMC11199556; doi:10.1038/s41467-024-48311-3)
Supplement: Supplementary file 1 — Supplementary Information [file 41467_2024_48311_MOESM1_ESM.pdf]

Landscape features support natural pest control and farm income when pesticide application is reduced

Supplementary information

Authors:

Ana Klinnert[1], Ana Luisa Barbosa[1], Rui Catarino[2], Thomas Fellmann[1], Edoardo Baldoni[1], Caetano Beber[1], Jordan Hristov[1], Maria Luisa Paracchini[2], Carlo Rega[3], Franz Weiss[2], Peter Witzke[4], Emilio Rodriguez-Cerezo[1]

Affiliation:

[1] European Commission, Joint Research Centre, C/Inca Garcilaso 3, 41092, Seville, Spain
[2] European Commission, Joint Research Centre, Via Enrico Fermi 2749, 21027, Ispra, Italy
[3] European Commission, Directorate General for Agriculture and Rural Development, Brussels, Belgium
[4]EuroCARE Bonn GmbH, Buntspechtweg 22, 53123 Bonn, Germany

1 LF-NPC:

1.1 Background on LF-NPC:

At its core, the LF-NPC indicator, developed by Rega et al. (2018) [1], combines the presence of diverse landscape elements – small woody features, grasslands, and forests – into an unified index to evaluate their collective contribution to support NPC. To emphasize its connection to landscape features, we refer to the developed NPC indicator as LF-NPC. The empirical foundation underpinning the approach of Rega et al. is derived from another study [2], which conducted a comprehensive analysis evaluating the effects of diverse landscape elements and their spatial arrangement within Semi Natural Habitats (SNH) on the abundance of natural enemies (see section 2.4). The results obtained on insect abundance were employed by Rega et al. (2018) to assign accordingly varying weights to landscape elements. Additionally, Rega et al. (2018) utilized a distance-weighted function to account for the influence of neighbouring cells on the target cell. Once normalized, in their study, the authors assigned to each agricultural pixel in Europe a LF-NPC score between 0 and 100, depending on the observed landscape composition. By applying zonal statistics to the LF-NPC map, we estimated the median score for each FADN region. A total of 133 FADN regions exist across the EU, with the smallest region located in Belgium at 133 square kilometres to the largest region in Sweden at 261,956 square kilometres. The distribution of these regions follows a skewed pattern, with the 75th percentile falling at around 38,900 square kilometres.

Table S1: FADN Regions: Area in (square kilometres). The table presents summary statistics depicting the size of FADN regions across Europe (n = 133). The majority of regions fall within the range of 31,500 to 38,900 square kilometres. Source data are available in the provided Source Data file.

Max	Min	Mean	25% Quantile	Median	75% Quantile
261,957	133	31,513	11,860	23,567	38,931

The LF-NPC score obtained for each FADN region was further normalized by the median score of its biogeographic region to enhance comparability between regions from different biogeographic regions. Biogeographic regions are categorized based on their biotas [3], encompassing the collection of all living organisms, including insects and microorganisms. Insects within a certain biota characterizing a biogeographic region may have different landscape requirements. Consequently, comparing the regional score of LF-NPC from different biogeographic regions could be misleading. To facilitate meaningful comparisons, a region's LF-NPC score should be normalized by the median LF-NPC score of its corresponding biogeographic region. This normalization process ensures that the LF-NPC values are comparable across regions, irrespective of their biogeographic context. This process results in LF-NPC scores ranging from 0 to a maximum of 2.7 across the EU (see Figure S1). Most regions fall within the range of 0 to 1.5, with the EU average standing at 0.9.

Figure S1: Regional LF-NPC score distribution. LF-NPC scores for each region (NUTS2) were obtained using zonal statistics. The mean score across all regions in Europe is depicted as a red dotted line. Total sample size: n = 326. Source data are provided as a Source Data file.

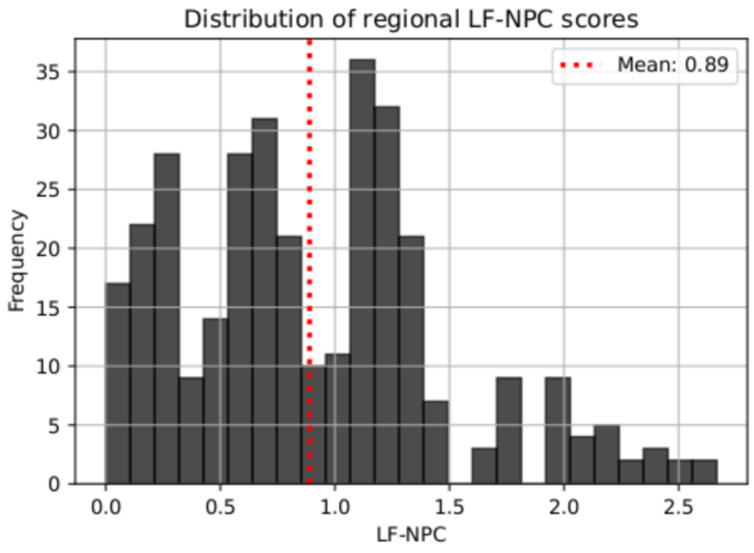

Table S2 offers a summary of pixel distribution across various quantiles and landscape elements for three regions characterized by different LF-NPC scores. This table aims to give a clearer understanding of the differences between regions with high and low LF-NPC scores. The table shows the percentage

distribution of pixels corresponding to different scores, as well as the relative abundance (in percentage terms) of each landscape element within each region, relative to the total regional area. Notably, the region of Lorrain, with a LF-NPC score of 2.66, has the highest percentage (92% = 0.3 +0.06 + 054 +0.02) of its area devoted to landscape elements contributing to the LF-NPC calculation.

Table S2: Proportion of LF-NPC Pixels in Specific Range and Landscape Features. The table presents the distribution of LF-NPC pixels across different score ranges (0-25, 25-50, 50-75, 75-100), relative to the total number of LF-NPC pixels in each region. It also shows the percentage of landscape features (woody areal, grasslands, forests) with respect to the total area of the region. The examples provided are for three distinct regions with varying LF-NPC scores. Source data are provided as a Source Data file.

	Pays de la Loire	Baden-Wuerttenberg	Lorrain
LF-NPC score	0.26	1.33	2.66
% of LF-NPC Pixel 0-25	0.90	0.66	0.39
% of LF-NPC Pixel 25-50	0.09	0.28	0.40
% of LF-NPC Pixel 50-75	0.01	0.06	0.21
% of LF-NPC Pixel 75-100	0.00	0.00	0.01
% Herbaceous Areal	0.14	0.14	0.30
% Woody Areal – Edge	0.05	0.07	0.06
% Woody Areal – Interior	0.11	0.51	0.54
% Woody Linear	0.01	0.02	0.02

1.2 Further refinements made to LF-NPC:

To further refine the LF-NPC indicator to the approximate location of organic farms, we exclude from our regional LF-NPC estimation, pixels categorized as intensive agriculture [3]. Organic farms are less likely to be concentrated in high-intensity agricultural areas and, therefore, should not be affected by the LF-NPC potential of those areas. Instead, we focus solely on pixels categorized as low and medium intense, which are likely to harbour a higher proportion of organic farms. By recalculating the LF-NPC score for each region using the agricultural intensity categories as a mask on top of the LF-NPC map, we obtain a regional LF-NPC score specific to low and medium intense agricultural areas. This modification results in improved estimations for some crops, as measured by the correlation coefficient and significance value (p-value) obtained from a simple linear regression (see Table S3). Hence, these results further strengthen the validity of our initial results obtained using the raw LF-NPC map[4].

Moreover, we pursued a further refinement of the LF-NPC indicator by computing crop-specific LF-NPC values per region using a detailed European crop-mask [5]. While this crop-mask pertains specifically to 2018 and thus may not perfectly align with the FADN yield observations used, spanning from 2010 to 2017, we employed an upscaling technique to transform the 10-meter crop mask of 2018

into a 10-km resolution map. Each 10x10 km pixel was assigned a value of 1 if the crop was present, and 0 otherwise. This map was then used as a crop-mask on top of the LF-NPC map to derive crop-specific LF-NPC estimates. Despite a loss in precision, this broader crop mask offers increased validity across multiple years, enabling its utilization for earlier periods. In essence, while a pixel classified as wheat in 2018 may not necessarily have been wheat in the years prior, the aggregate number of pixels identified as wheat in 2018 within a 10-kilometer square varies less across years compared to solely focusing on the identity of a single 10x10 meter pixel.

By employing this crop-mask on top of the previously refined LF-NPC map, and recalculating the median score for each region, crop-specific LF-NPC scores are obtained. This methodology is expected to be more accurate for the purpose of our study. For instance, if barley in a particular region is confined to a specific area within that region, then only the LF-NPC pixels within that precise area should matter to explain the yield gap for barley between organic and conventional farming. The re-estimation results in LF-NPC scores ranging from 0 to a maximum of 2.9 across the EU (see Figure S2). Once again, most regions fall within the range of 0 to 1.5, with the EU average remaining at 0.9 (see Figure S2). Upon evaluating the performance of the newly estimated crop-specific LF-NPC score in explaining yield gaps, we found that its efficacy only increased for a select few crops, notably rye and potatoes. For the remaining crops, the p-values and correlation coefficients remained relatively unchanged (see Table S3).

Figure S2: Crop-specific LF-NPC score distribution. Crop-specific LF-NPC scores for each region (NUTS2) were obtained using zonal statistics. The mean score across all regions is depicted as a red dotted line. Total sample size: n = 326. Source data are provided as a Source Data file.

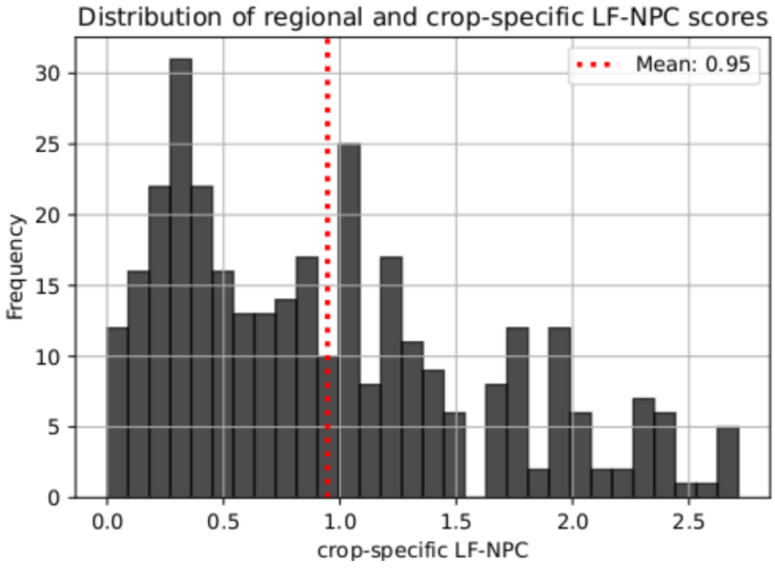

Table S3: LF-NPC. Performance evaluation between variants of the LF-NPC indicator: LF-NPC normalized by biogeographic region (LF-NPC), LF-NPC additionally tailored to low- and medium-intensity agricultural areas (LF-NPC - organic) and to the right crop-specific LF-NPC tailored to low- and medium-intensity agricultural areas (Crop-specific LF-NPC - organic). The relationship between each variant of LF-NPC and yield gaps is shown in this table. It includes the Pearson correlation coefficient, the coefficient derived from a simple linear regression, and the associated P-value of the LF-NPC coefficient, calculated using a two-sided paired t-test. Sample size: All crops: n = 326; Barley: n = 59; Wheat: n = 55; Oats: n =47; Peas: n = 36; Corn: n = 28; Rye: n = 25; Potatoes: n = 22; Legumes: n = 21; Durum Wheat: n = 20; Fodder Corn: n = 13. Source data are provided as a Source Data file.

	LF - NPC			LF-NPC - organic			Crop-specific LF-NPC - organic		
Crop	Correlation, ρ	Coefficient - LR	p-value	Correlation, ρ	Coefficient - LR	p-value	Correlation, ρ	Coefficient - LR	p-value
Barley	0.17	3.79	0.19	0.3	6.09	0.02	0.26	5.63	0.05
Wheat	0.08	1.71	0.54	0.24	4.29	0.08	0.24	4.2	0.08
Oats	0.08	1.6	0.59	0.32	5.77	0.03	0.23	3.52	0.13
Peas	0.21	4.97	0.22	0.2	4.22	0.24	0.2	4.22	0.24
Corn	0.28	4.73	0.15	0.12	2.08	0.54	0.14	2.32	0.49
Rye	0.13	3.74	0.52	0.03	0.81	0.9	0.05	1.53	0.81
Potatoes	0.27	9.54	0.13	0.26	7.58	0.24	0.4	10.69	0.06
Legumes	0.09	1.15	0.7	0.32	3.47	0.15	0.32	3.47	0.15
Durum Wheat	0.14	1.76	0.55	0.12	1.39	0.61	0.24	2.69	0.32
Fodder Corn	0.54	14.74	0.06	0.32	7.17	0.28	0.33	7.22	0.28

Table S4: Mixed Effect Results. Left estimate: Results obtained from only allowing the intercept to vary. P-value for intercept and coefficient 1.20e-09 and 1.82e-05 respectively. Right estimate: Results obtained from allowing intercept and independent variable (LF-NPC) to vary. P-value for intercept and coefficient 1.20e-09 and 1.82e-05 respectively. Reported p-value calculated using a two-sided paired t-test. Total observations used n = 326. Source data are provided as a Source Data file.

	estimate			estimate		
Predictors	*Estimates*	*CI*	*p*	*Estimates*	*CI*	*p*
(Intercept)	-19.33	-22.70 – -15.97	**<0.001**	-19.27	-22.77 – -15.76	**<0.001**
LF-NPC	4.36	2.39 – 6.33	**<0.001**	4.31	2.33 – 6.28	**<0.001**
**Random Effects**						
σ^2	144.86			144.79		
τ_{00}	15.12 $_{crop}$			17.52 $_{crop}$		
τ_{11}				0.09 $_{crop.median\_biogeo\_norm}$		
ρ_{01}				-1.00 $_{crop}$		
ICC	0.09					
N	10 $_{crop}$			10 $_{crop}$		
Observations	326			326		
Marginal R^2 / Conditional R^2	0.052 / 0.141			0.055 / NA		

2 Yield gap estimation

2.1 Crop selection:

Yield gap estimations were limited to crops and regions where at least 16 yield observations in both organic and conventional farming were available in the FADN dataset. Furthermore, the contribution of LF-NPC potential to yield gap was only analysed for crops with yield gap estimations available in more than 12 regions. These selection criteria resulted to the examination of LF-NPC contributions to yield gaps for ten crops: wheat, corn, barley, oats, peas, potatoes, rye, fodder corn, durum wheat and legumes. This selection ensured robust analysis by requiring:

- At least 16 observations of organic farming per crop in each region: This guarantees reliable estimates of regional yield gaps.
- At least 12 regional yield gap estimates across the EU: This allows us to avoid overfitting when analyzing the relationship between LF-NPC and yield gaps.

Table S5: Average yield gap between organic and conventional farming by crop (in %). Averages are derived from regional yield gap estimates obtained from regression model. Yield gap represent the difference in productivity between organic and conventional farming due to differences in pesticides use. Source data are provided as a Source Data file.

Legumes	-8.3
Durum Wheat	-8.6
Fodder Corn	-13.0
Corn	-13.0
Peas	-13.3
Oats	-13.9
Barley	-17.3
Potatoes	-17.3
Wheat	-22.2
Rye	-22.9

2.2 Model performance:

Although yield gaps in this study are not selected based on a threshold that defines their statistical significance (as explained below), we still check for homoscedasticity, normality, and serial correlation. To this purpose, we utilized results from all 904 models executed to derive 534 region-specific crop yield gaps. It is essential to highlight that for a particular crop within a region, multiple models and corresponding yield gaps can be generated across various specializations (TF8) (see Method section). These values are subsequently averaged to produce a single regional crop-specific estimate. Therefore, while the total count of models and yield gaps obtained is 904, the resulting averaged final crop-specific yield gaps amount to 534. Using robust estimation, 61% of the estimated p-values of the

organic dummy are significant at 95% confidence level, and 67% of the estimated p-values are significant at 90% confidence level. Setting the statistical significance at lower confidence levels (i.e., by allowing significant estimates having p-values larger than 0.1), leads to a notably higher number of yield gap estimations. The graph below shows the relationship between confidence levels and the share of statistically significant estimates. This nuanced exploration of significance levels contributes to a more comprehensive understanding of the robustness of our findings, acknowledging variations in confidence thresholds.

Figure S3: Share of significant estimates (organic dummy) across confidence thresholds. The x-axis represents the confidence level (1-α) as percentage, where α represents the p-value. The y-axis illustrates the percentage share of significant elements, i.e., the share of estimates associated with a p-value smaller than α. The graph shows the p-values of all region-crop-specialization yield gaps (n=904) used to obtain the aggregated 534 region-crop specific gaps. Source data are provided as a Source Data file.

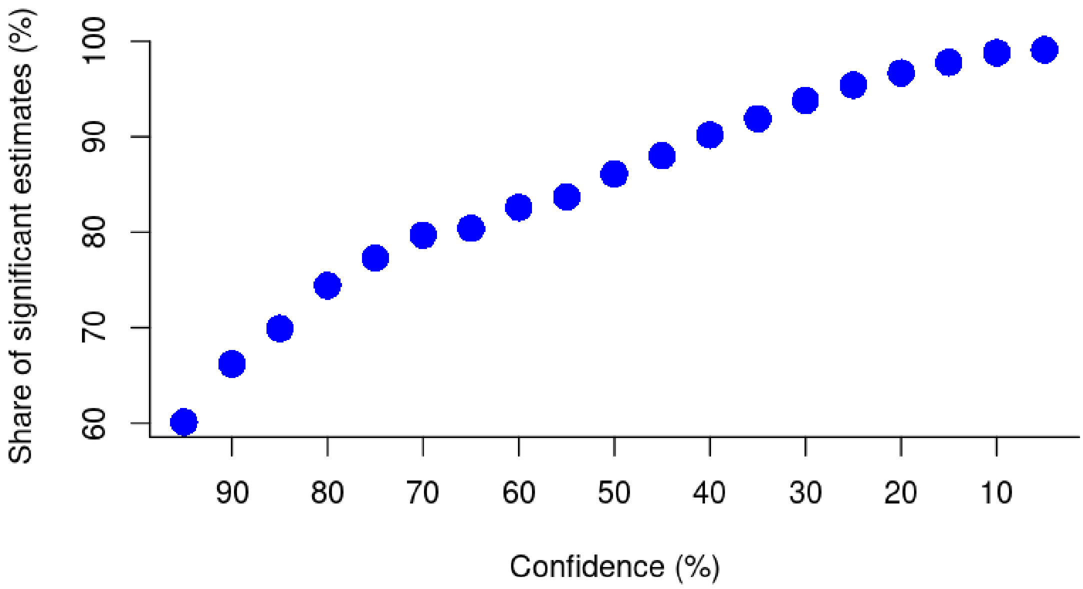

To further support the choice of not filtering by p-value, we perform a sensitivity analysis, which reveals that the consequences of our approach are limited in magnitude but beneficial for extending the analysis to larger geographical areas. In the accompanying scatterplots, we exclusively employ yield gap estimations with a significance level of 90%. Notably, the overall trend remains consistent across all crops, except for corn where the correlation between LF-NPC and yield gap disappears when focusing on the selected yield gaps. For the remaining crops, however, the positive overall correlation persists.

Figure S4: Sensitivity test LF-NPC versus yield gaps per crop. Each chart corresponds to a specific crop (see chart title), with the initial chart consolidating all crops. The x-axis denotes the LF-NPC score,

while the y-axis represents the yield gap between organic and conventional farming resulting from variations in pesticide usage. Sample sizes: All crops: n = 234; Barley: n = 44; Wheat: n = 49; Oats: n =33; Peas: n = 21; Corn: n = 14; Rye: n = 26; Potatoes: n = 18; Legumes: n = 13; Durum Wheat: n = 16. Source data are provided as a Source Data file.

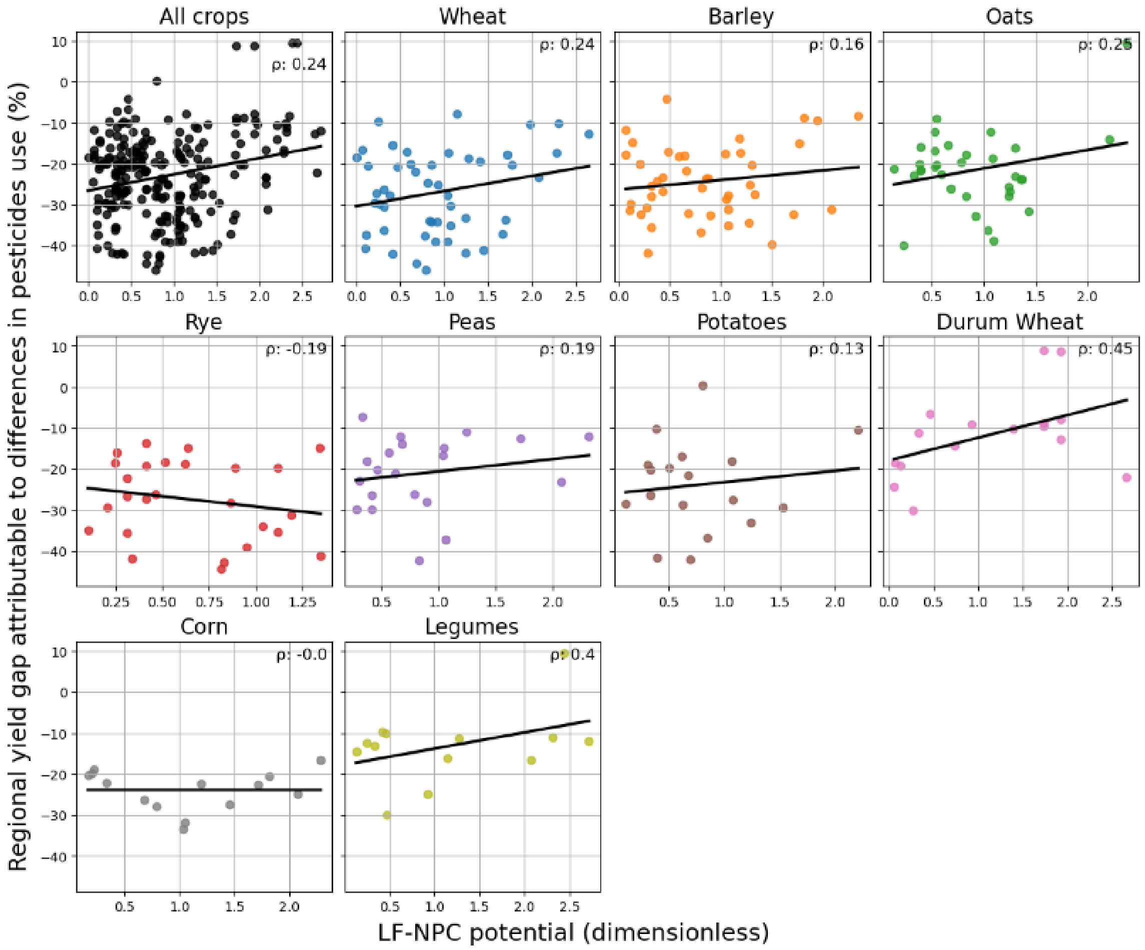

Despite not explicitly considering the statistical significance of coefficients, we provide evidence on the model diagnostics for the 904 yield gap estimates obtained. Once more, it is crucial to emphasize that these diagnostics must be derived from the entirety of the 904 yield gaps, each associated with an individual model. They cannot be derived from the averaged final 534 region- and crop-specific yield gaps.

We use the Breusch-Pagan test for heteroscedasticity, the Breusch-Godfrey test of serial correlation test and the Shapiro test for testing the normality of residuals after the Ordinary Least Squares (OLS) estimation.

- Heteroscedasticity: 35% of the estimated models have a p-value of the Breusch-Pagan test larger than 0.1 (residuals are homoscedastic), increasing to 42% by setting the 0.05 p-value threshold.
- Serial correlation: 62% of the estimated models have a p-value of the serial correlation test higher than 0.1, reaching 71% considering a p-value of 0.05.
- Normality: around 13% of the estimated models show a higher than 0.1 p-value (normality) for the Shapiro test, increasing to 16% at a 0.05 p-value threshold.

Overall, these tests show that the assumption of normality of residuals is often not satisfied, homoscedasticity is satisfied in a share of models ranging between 35 and 42%, while most models do not show serial correlation in their residuals.

Regarding the non-normality of residuals, in this case they do not pose a threat to the consistency of OLS estimates. Even in the absence of normality, OLS would still be the best linear unbiased estimator (BLUE) according to the Gauss-Markov theorem [6]. Normality is only desirable as it implies that OLS is efficient also against non-linear estimators [7]. In our application, the use of non-linear estimator is risky: these are typically based on maximization routines that have to be led to convergence. Even when a non-linear estimator has an analytical solution (for example, Maximum Likelihood Estimation (MLE) or Generalized Method of Moments (GMM)), in practice, this solution has to be reached numerically. In our application, where we estimate several hundreds of models, it is difficult to control for convergence and for the presence of multiple maxima in each optimization. Moreover, efficiency of estimates of non-linear estimators (for example of MLE) can only be guaranteed asymptotically while in small samples other estimators may have greater concentration around the true parameter value [8]. In this context, the use of OLS in this study is, therefore, the safer choice.

Furthermore, as additional diagnostics on residuals, we explore graphically their distribution after pooling all the regional errors together and displaying them by crop. As testing for normality in large samples is especially complex and unreliable, we test it visually using histograms and a normality plot, or QQ plot.

Figure S5: Error distribution per crop. Frequency histogram of errors obtained from comparing the estimated yield gap from the regression models to the declared yield gap under FADN. The number of farm-level observations per crop are as follows: Barley: n=209,277; Fodder corn: n=38,571; Maize: n=77,324; Legumes: n=16,141; Oats: n=71,723; Peas: n=48,575; Potatoes: n=97,396; Rye: n=64,332; Common Wheat: n=262,838; Durum Wheat: n=37,368. Source data are provided as a Source Data file.

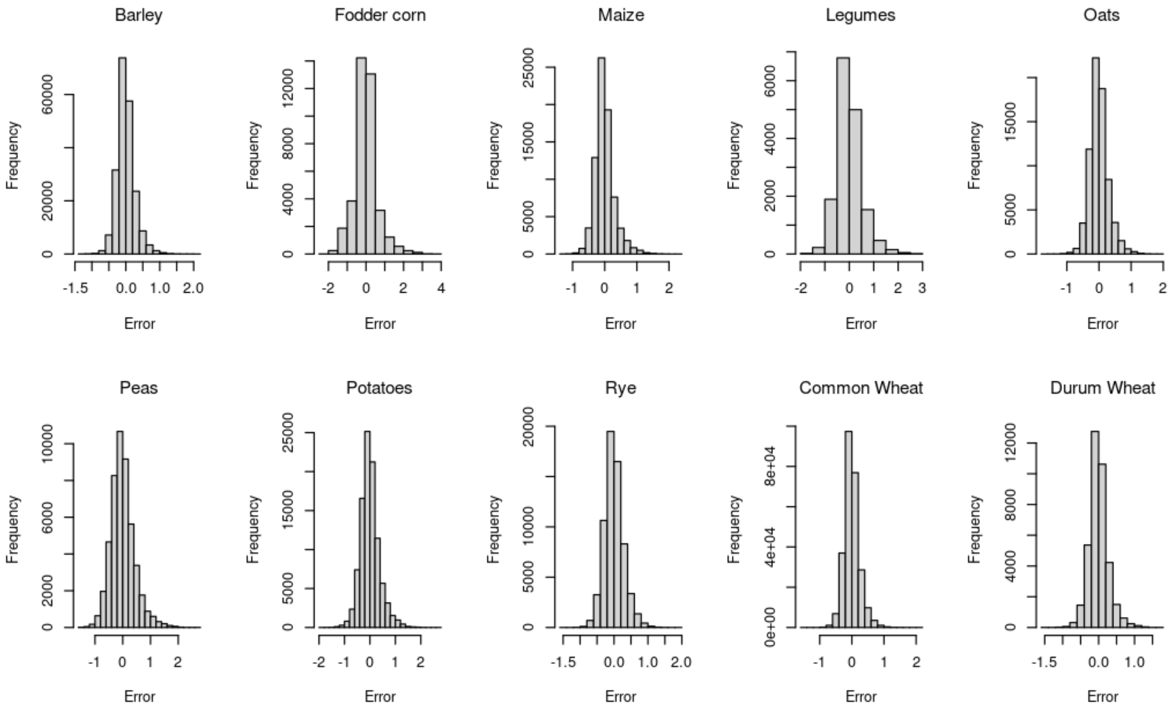

Besides the case of fodder corn, the histograms present a certain degree of symmetry.

Figure S6: QQ- plots of errors per crop. QQ-plots of errors obtained from comparing estimated yield gap from the regression models to the declared yield gap under FADN. The number of observations per crop are as follows: Barley: n=209,277; Fodder corn: n=38,571; Maize: n=77,324; Legumes: n=16,141; Oats: n=71,723; Peas: n=48,575; Potatoes: n=97,396; Rye: n=64,332; Common Wheat: n=262,838; Durum Wheat: n=37,368. Source data are provided as a Source Data file.

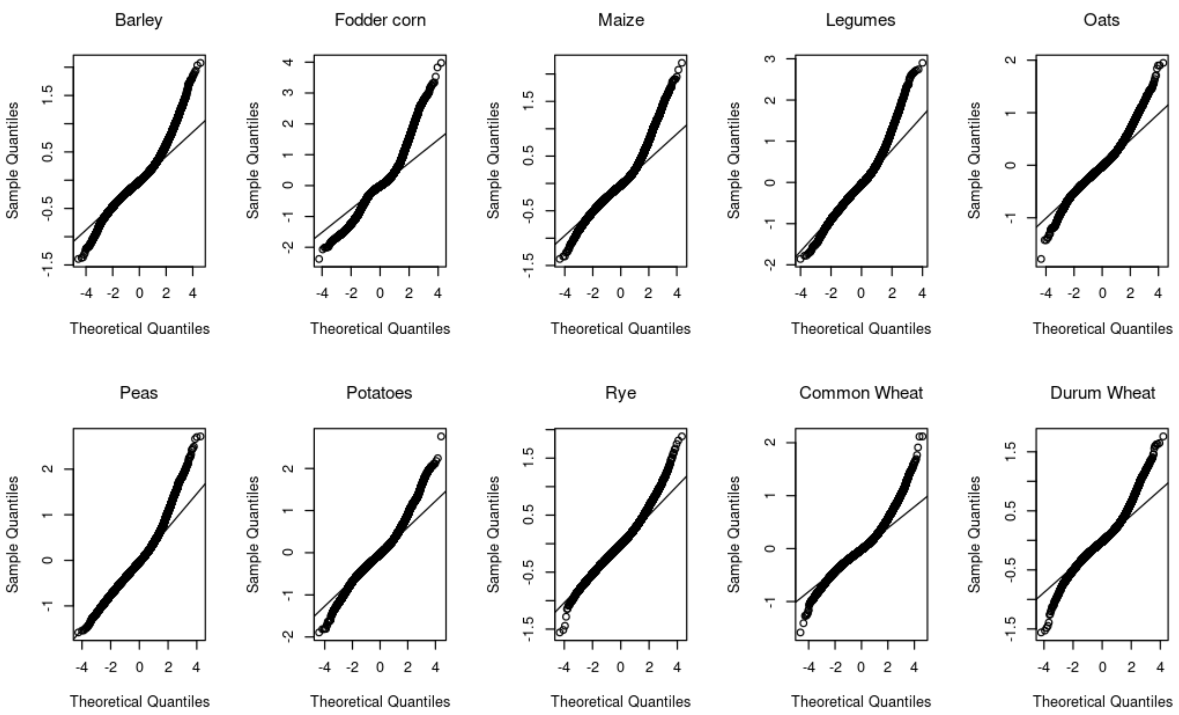

With respect to the QQ plots, if the error distributions were normally distributed, points would align along the bisecting line of the plot. In the case of our distributions, the comparison of sample quantiles (y-axis) with theoretical quantiles (x-axis) shows the presence of some skewness and heavier-than-normal tails. In the case of fodder corn, legumes and peas, distributions present some skewness to the right while the rest of the distribution is close to a normal distribution.

Moreover, we check on possible issues due to leverage. Leverage is a measure of how far away the independent variable values of an observation are from those of other observations. High-leverage points have the potential of being influential points, but they are not necessarily such [9]. Leverage is based on the hat matrix $\mathrm{H} = \mathrm{X}\left(\mathrm{X}^{\mathrm{T}}\mathrm{X}\right)^{-1}\mathrm{X}^{\mathrm{T}}$, where X is the matrix of explanatory variables used in a model. The diagonal elements of the hat matrix, h_{ii}, can be defined as a weighted distance between observation x_i to the mean of all x_i's. A rule of thumb to identify leverage points is to identify the points whose leverage value exceeds two/three times the mean average leverage value. We provide statistics for both cases. In the case of the first threshold (two times the mean average leverage value), the average share of influential observations for models is 5.7%. In the case of the second threshold

(3 times the average leverage value), the average share of influential observations for all models is 2.4%.

3 Simulation under the partial equilibrium model CAPRI:

For the economic evaluation of LF-NPC we simulated two different scenarios: One scenario which does not consider feedbacks from trade and related price effects from global agri-food markets, and a second scenario where these price and market feedbacks are considered constant. Both scenarios were run using the latest available Common Agricultural Policy Regionalised Impact Modelling System (CAPRI) version at the time of the analysis. This allowed making use of an updated database and using 2017 as the base year.

When using the model without market feedback, the exogenous yield changes introduced trigger endogenous responses depending on the relative profitability between crops and price elasticities [10]. When running the CAPRI model with the market feedback activated, the endogenous reaction depends on an iterative process triggered by supply and price changes in the global and local markets, following the micro economic theory between supply and demand until an equilibrium is reached between supply and demand and the market balance is achieved.

Both scenarios show a positive relative contribution of LF-NPC potential to agricultural income. As our simulation mainly focuses on arable crops, the agricultural income analysed is the one of utilized agricultural area. In the scenario without international market and price feedback, a one-unit increase in the LF-NPC potential results in a 4.4% improvement in agricultural income per hectare (Figure S4). However, we observe that overall agricultural income decreases compared to the baseline (see y-axis of Figure S4) due to generally lower yields as a consequence of reduced pesticides use. Only regions with a high LF-NPC potential are able to minimize the impact on income and stay close to what would have been their income under the business-as-usual baseline. These results are in line with the general logic of running a partial equilibrium model without allowing market feedback to increase prices as a result of lower production, i.e. constant prices and lower yields will lead to lower farm income. In contrast, when running a scenario where we allow for market feedback, prices increase as a result of lower production. The higher prices imply that regions with a below-average LF-NPC potential are shown to increase their income compared to the baseline. However, in our study we are not interested in income changes due to price adjustments. We focus on the relative change in income with respect to varying LF-NPC scores.

Figure S7: LF-NPC potential versus income changes in percentage. The scatterplot shows the relation between LF-NPC score and income changes. The red line represents a simple linear fitted regression line. Each point represents a region's (NUTS2) LF-NPC score (x-axis) and the related income change (y-axis). The text box in the lower right corner shows the equation obtained from a simple linear regression of LF-NPC against income changes, the p-value of the coefficient LF-NPC calculated using a two-sided paired t-test, and the adjusted R-square. Sample size: n = 211. Source data are provided as a Source Data file.

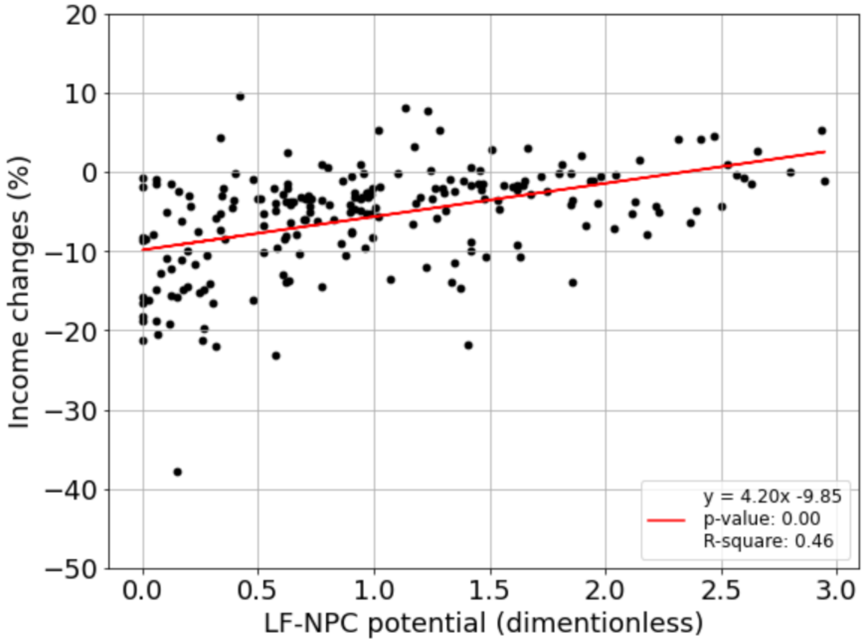

Furthermore, we focused in this study on the income of utilized agricultural area instead of looking at crop-specific income changes, as structural characteristics, such as the cost structure at regional level, can introduce important variations in the income that are not related to the LF-NPC potential. When looking at the overall income from utilized agricultural area, the crop-specific cost structures are less important and hence allow for a more direct relation between LF-NPC and income changes expressed in percentage changes relative to the baseline scenario. Similarly, it is important to note that when comparing yield changes to crop-specific revenue changes, the impact is more direct, as revenue is not influenced by production costs, unlike income, which considers both revenue and cost.

Supplementary Code

All analysis code and output are available through our GitHub project site [11] https://github.com/anakl/LF-NPC and are released on Zenodo (DOI: 10.5281/zenodo.11040631.).

The GitHub folder contains the following files:

1. revision_code_yieldgaps_clean.R: R script estimating the yield gap between organic and conventional farming due to differences in pesticides use. Please be aware that this script requires access to farm-level FADN data for its execution, which under the EU GDPR regulation cannot be shared by any circumstances to third parties. Nevertheless, to maximize transparency we include the R-script used to calculate the yield gaps. The R-script consists of two main functions: One function calculates the coefficients of the covariates influencing yields and a second one converts the coefficient to a yield gap. The script aligns with the methodology described under "Yield gaps between organic and conventional farming" within the method section.
2. mixed_effect_model.R: R script running the mixed effect model capturing the impact of LF-NPC on yield gap. Please ensure you have the necessary data (“input_npc_yield_gap_data.xlsx”) saved in the same directory as this R script. This will allow you to execute the script without any problems and obtain the same results as presented in the results file called “output_mixed_effect.xlsx”.
3. input_npc_yield_gap_data.xlsx: Excel file containing the data on LF-NPC and yield gaps per crop at regional level (NUTS2). This data is inserted into the mixed effect model “mixed_effect_model.R”.
4. output_mixed_effect.xlsx: Excel file containing the output from ”mixed_effect_model.R”.
5. results_NPC_income.xlsx: Excel file containing the data on a region’s LF-NPC scores and income (baseline and future simulation). The income has been estimated by the CAPRI modelling system. The model is open-sourc - see: https://www.capri-model.org/doku.php?id=capri:install.
6. Source File.xlsx: This Excel file contains the data used to produce all figures and tables of the manuscript and the supplementary material.
7. variation_FADN_regions.xlsx: Excel file containing information on LF-NPC and yield gap (per crop) variations within FADN regions. The variation is captured using various metrics: minimum, maximum, 25th and 75th percentiles, mean, median, and standard deviation. Statistics on the LF-NPC scores are obtained taking into account all agricultural pixels within each region. Yield gap data is presented per crop, with dedicated tabs for each individual crop.

Supplementary references

1. Rega C, Bartual AM, Bocci G, et al. A pan-European model of landscape potential to support natural pest control services. *Ecol Indic*. 2018;90. doi:10.1016/j.ecolind.2018.03.075
2. Bonato M, Martin EA, Cord AF, Seppelt R, Beckmann M, Strauch M. Applying generic landscape-scale models of natural pest control to real data: Associations between crops, pests and biocontrol agents make the difference. *Agric Ecosyst Environ*. 2023;342. doi:10.1016/j.agee.2022.108215
3. Morrone JJ. The spectre of biogeographical regionalization. *J Biogeogr*. 2018;45(2):282-288. doi:https://doi.org/10.1111/jbi.13135
4. Rega C, Short C, Pérez-Soba M, Luisa Paracchini M. A classification of European agricultural land using an energy-based intensity indicator and detailed crop description. *Landsc Urban Plan*. 2020;198. doi:10.1016/j.landurbplan.2020.103793
5. d'Andrimont R, Verhegghen A, Lemoine G, Kempeneers P, Meroni M, van der Velde M. From parcel to continental scale – A first European crop type map based on Sentinel-1 and LUCAS Copernicus in-situ observations. *Remote Sens Environ*. 2021;266. doi:10.1016/j.rse.2021.112708
6. Hill RC, Griffiths WE, Lim GC (Guay C. *Principles of Econometrics / R. Carter Hill, William E. Griffiths, Guay C. Lim.*; 2018.
7. Hayashi F. *Econometrics*. Princeton University Press; 2011. https://books.google.es/books?id=QyIW8WUIyzcC
8. Pfanzagl J. *Parametric Statistical Theory*.; 1994. doi:10.1515/9783110889765
9. Cardinali C. Observation influence diagnostic of a data assimilation system. In: *Data Assimilation for Atmospheric, Oceanic and Hydrologic Applications (Vol. II)*. ; 2013. doi:10.1007/978-3-642-35088-7_4
10. Jansson T, Heckelei T. Estimating a Primal Model of Regional Crop Supply in the European Union. *J Agric Econ*. 2011;62(1). doi:10.1111/j.1477-9552.2010.00270.x
11. Klinnert A, Barbosa AL, Catarino R, et al. *Landscape Features Support Natural Pest Control and Farm Income When Pesticide Application Is Reduced*. https://github.com/anakl/LF-NPC. doi:10.5281/zenodo.11040631. 2024
