# Supplementary material for: Landscape features support natural pest control and farm income when pesticide application is reduced
Source: Nat Commun. 2024 Jun 25;15:5384. doi: 10.1038/s41467-024-48311-3 (PMC11199556; doi:10.1038/s41467-024-48311-3)
Supplement: Supplementary file 2 — Reporting Summary [file 41467_2024_48311_MOESM2_ESM.pdf]

nature portfolio

Corresponding author(s): Ana Klinnert

Last updated by author(s): Apr 20, 2024

Reporting Summary

Nature Portfolio wishes to improve the reproducibility of the work that we publish. This form provides structure for consistency and transparency in reporting. For further information on Nature Portfolio policies, see our Editorial Policies and the Editorial Policy Checklist.

Statistics

For all statistical analyses, confirm that the following items are present in the figure legend, table legend, main text, or Methods section.

n/a	Confirmed	
☐	☒	The exact sample size (*n*) for each experimental group/condition, given as a discrete number and unit of measurement
☐	☒	A statement on whether measurements were taken from distinct samples or whether the same sample was measured repeatedly
☐	☒	The statistical test(s) used AND whether they are one- or two-sided<br>*Only common tests should be described solely by name; describe more complex techniques in the Methods section.*
☐	☒	A description of all covariates tested
☐	☒	A description of any assumptions or corrections, such as tests of normality and adjustment for multiple comparisons
☐	☒	A full description of the statistical parameters including central tendency (e.g. means) or other basic estimates (e.g. regression coefficient) AND variation (e.g. standard deviation) or associated estimates of uncertainty (e.g. confidence intervals)
☐	☒	For null hypothesis testing, the test statistic (e.g. *F*, *t*, *r*) with confidence intervals, effect sizes, degrees of freedom and *P* value noted<br>*Give P values as exact values whenever suitable.*
☒	☐	For Bayesian analysis, information on the choice of priors and Markov chain Monte Carlo settings
☒	☐	For hierarchical and complex designs, identification of the appropriate level for tests and full reporting of outcomes
☒	☐	Estimates of effect sizes (e.g. Cohen's *d*, Pearson's *r*), indicating how they were calculated

Our web collection on statistics for biologists contains articles on many of the points above.

Software and code

Policy information about availability of computer code

Data collection	In this research project we have not been involved in data collection. The work fully relies on already collected data from other research projects, which in the "Data Availability" section have been referenced.
Data analysis	R (version 4.3.3): Open- source software environment designed for statistical computing and data analysis.<br>--<br>Microsoft Excel (Professional Plus 2016): Spreadsheet editor developed by Microsoft. This software has been used to store the data and provide the Source File.<br>--<br>CAPRI (TRUNK version - October 2022): CAPRI is an agro-economic model employed for evaluating how agricultural, trade, and environmental policies affect agriculture. This model furnishes outcomes at both regional levels and across a spectrum of economic and environmental factors. For more information on CAPRI see https://www.capri-model.org/.

For manuscripts utilizing custom algorithms or software that are central to the research but not yet described in published literature, software must be made available to editors and reviewers. We strongly encourage code deposition in a community repository (e.g. GitHub). See the Nature Portfolio guidelines for submitting code & software for further information.

Data

Policy information about availability of data

All manuscripts must include a data availability statement. This statement should provide the following information, where applicable:

- Accession codes, unique identifiers, or web links for publicly available datasets
- A description of any restrictions on data availability
- For clinical datasets or third party data, please ensure that the statement adheres to our policy

Data availability
1. FADN: https://agriculture.ec.europa.eu/data-and-analysis/farm-structures-and-economics/fadn_en. The raw FADN data are protected and are not available due to data privacy laws: "According to Regulation (EC) No 1217/2009 the data are covered by strict confidentiality rules and can only be used to meet the needs of the common agricultural policy. For example, they cannot be used by authorities for tax or compliance purposes."

2. LF-NPC as per Rega et al 2018 is available at: https://www.sciencedirect.com/science/article/pii/S1470160X18302309

3. European crop mask 2018 is available at:
https://www.sciencedirect.com/science/article/pii/S0034425721004284

4. Classification of European agricultural land crop-management systems is available at:
https://www.sciencedirect.com/science/article/pii/S0169204618314440

5. Source Data: All data generated in this study are provided in the Supplementary Source Data file provided with this paper and are accessible through the GitHub repository: https://github.com/anakl/LFNPC.

Research involving human participants, their data, or biological material

Policy information about studies with human participants or human data. See also policy information about sex, gender (identity/presentation), and sexual orientation and race, ethnicity and racism.

Reporting on sex and gender	N/A
Reporting on race, ethnicity, or other socially relevant groupings	N/A
Population characteristics	N/A
Recruitment	N/A
Ethics oversight	N/A

Note that full information on the approval of the study protocol must also be provided in the manuscript.

Field-specific reporting

Please select the one below that is the best fit for your research. If you are not sure, read the appropriate sections before making your selection.

- [ ] Life sciences
- [ ] Behavioural & social sciences
- [x] Ecological, evolutionary & environmental sciences

For a reference copy of the document with all sections, see nature.com/documents/nr-reporting-summary-flat.pdf

Ecological, evolutionary & environmental sciences study design

All studies must disclose on these points even when the disclosure is negative.

Study description	The study analyses the relationship between natural pest control induced by landscape features (LF_NPC) and yield gap differences between intensive (conventional) and extensive (organic) farm practices. In a first stage, the yield haps are estimated using data from the EU Farm Accountancy Data Network (FADN). In a second step, these yield gaps are confronted to regional median estimates of LF-NPC with the aim of estimating the contribution of LF-NPC in reducing the yield hap between intensive and less intensive farm practices.
Research sample	FADN surveys annually a large sample of agricultural holdings (around 80,000) across all EU Member States covering a wide range of farm types and sizes. It focuses on the collection of production, economic, financial and environmental data of agricultural holdings and is meant to be a representation of EU farms. We have selected this sample, as it is one of the most extensive agricultural and economic databases covering all EU Member States.<br><br>The LF-NPC index is provided by Rega et al 2018 at 100 meter resolution for all Europe. The index is based on a spatially-explicit

	model to map and assess the landscape potential sustaining natural pest control (LF-NPC). The index ranges between 0 and 100, with 100 indicating the maximum LF-NPC. This data represents across the EU the LF-NPC potential of a 100x100 meter pixel. To the knowledge of the authors it is the only source to provide LF-NPC potential across Europe and hence has been chosen for this study.
Sampling strategy	We did not use a sampling strategy in this study, as all data was of value and hence used. We only excluded data for statistical robustness as explained under the section "Data exclusions".
Data collection	This study did not have to engage in data collection as the data was already provided by the FADN or through Rega et al. 2018.
Timing and spatial scale	The FADN data covers farms across the entire European Union. To match closely the time scale of the LF-NPC index we focused on the years 2010 to 2017. The natural pest control index is available across all the European continent at 100 meter resolution. The time scale of the underlying layers used to create the index vary between 2012 and 2015.
Data exclusions	Yield gaps between organic and conventional farming:<br>To estimate the yield gap between organic and conventional farming due to differences in pesticides use, we excluded the upper and lower one percentile of yield observations, to get rid of extreme and unfeasible values. Furthermore, to ensure statistical robustness, FADN regions with fewer than 16 organic farms cultivating a specific crop were excluded from the analysis. Finally it is also worth to mention, that we have have excluded yield observations with implausible biophysical values.<br><br>Contribution of LF-NPC potential to agricultural production:<br>Similarly, in the mixed effect model only crops for which more than 12 observations were present, were included.
Reproducibility	Yield gaps between organic and conventional farming:<br>The input data, sourced from FADN used to calculate the yield gaps at regional level cannot be shared externally due to data privacy restrictions. Nevertheless, to maximize transparency, we have included the R script used for estimating the yield gaps, which has allowed for internal replication.<br><br>Contribution of LF-NPC potential to agricultural production:<br>The results of the mixed-effect model, which assesses the influence of LF-NPC on yield gaps, are reproducible using the provided data consisting of yield gaps and the regional-level natural pest control index, along with the accompanying R script.
Randomization	Yield gaps between organic and conventional farming:<br>The yield gap estimations were conducted per FADN region with each region representing a distinct geographical area reported within the FADN dataset. It's important to note that farm-specific coordinates, which could provide precise farm locations, were not available for our analysis. Additionally, in selecting covariates for our analysis, we carefully considered variables that, based on both established theory and common knowledge, were likely to influence the yield gap between intensive (conventional) and less intensive (organic) farming practices. Detailed explanations for the inclusion of each covariate are provided in the methodology section.<br><br>Contribution of LF-NPC potential to agricultural production:<br>No random sampling or selection of covariates took place during this process.
Blinding	Blinding is not of relevance in this process as they data was not collected by the authors of the study.

Did the study involve field work? ☐ Yes ☒ No

Reporting for specific materials, systems and methods

We require information from authors about some types of materials, experimental systems and methods used in many studies. Here, indicate whether each material, system or method listed is relevant to your study. If you are not sure if a list item applies to your research, read the appropriate section before selecting a response.

Materials & experimental systems

n/a	Involved in the study
☒	☐ Antibodies
☒	☐ Eukaryotic cell lines
☒	☐ Palaeontology and archaeology
☒	☐ Animals and other organisms
☒	☐ Clinical data
☒	☐ Dual use research of concern
☒	☐ Plants

Methods

n/a	Involved in the study
☒	☐ ChIP-seq
☒	☐ Flow cytometry
☒	☐ MRI-based neuroimaging

Plants

Seed stocks	N/A
Novel plant genotypes	N/A
Authentication	N/A
